# Optimization of processing parameters of 50 % aged paddy rice replace of corn in feed formula: effects of grinding sieve opening, conditioning temperature and time on the growth performance, meat quality and serum biochemical parameters in broilers

**DOI:** 10.1016/j.psj.2025.105019

**Published:** 2025-03-10

**Authors:** Jie Yang, Yue Shi, Xing Li, Junguo Li, Xiaohan Shi, Chi Zhang, Manqi Wang, Yuchang Qin, Jiaqi Zhang

**Affiliations:** a Institute of Feed Research, Chinese Academy of Agricultural Sciences, Beijing 100081, China; b Hubei Key Laboratory of Animal Nutrition and Feed Science, Wuhan Polytechnic University, Wuhan 430023, China; c Institute of Animal Science, Chinese Academy of Agricultural Sciences, Beijing 100193, China

**Keywords:** Aged paddy rice, Sieve openings, Conditioning temperature, Conditioning time, Growth performance

## Abstract

This study aimed to evaluate the effects of different grinding sieve openings (SO), conditioning temperatures (CTP), and conditioning times (CTM) on feed processing performance, growth performance, meat quality, and serum biochemical indices of broiler chickens fed a diet in which 50 % of corn was replaced with aged paddy rice. A total of 960 white-feathered broiler chickens were randomly divided into 8 groups, with the experiment designed to test 2 SO sizes (2.0 mm and 2.5 mm), 2 CTPs (75 °C and 85 °C), and 2 CTMs (90 s and 180 s). The results showed that the 2.0 mm SO and 180 s CTM significantly improved pellet hardness and the pellet durability index (PDI) (*P* < 0.05). During the early stages of the experiment, when the broilers digestive systems were not fully developed, a 2.5 mm SO significantly reduced the feed conversion ratio (FCR) (*P* < 0.001), while 75 °C CTP significantly increased average daily gain (ADG) and body weight (BW) at day 21 (*P* < 0.05). Additionally, 75 °C CTP significantly reduced the cecum organ weight ratio (*P* < 0.05). In terms of meat quality, a 2.5 mm SO significantly reduced drip loss and cooking loss at 48 h and 72 h (*P* < 0.01, *P* < 0.01, *P* < 0.05), while a 90 s CTM significantly reduced drip loss and cooking loss at 24 h (*P* < 0.05, *P* < 0.01). Furthermore, the combination of 2.5 mm SO and 90 s CTM significantly increased serum urea nitrogen (BUN) levels (*P* < 0.05). Considering the optimization of both growth performance and meat quality, the combination of 2.5 mm SO, 75 °C CTP, and 90 s CTM is recommended for optimizing aged paddy rice feed processing and enhancing broiler growth performance and meat quality.

## Introduction

The volatility of corn prices and tightening global supplies, driven by escalating competition between human food and animal feed demands, has intensified the search for sustainable alternatives in poultry feed production (Loy and Lundy, 2019). For over six decades, broiler feed manufacturing has relied on standardized conditioning (75–85 °C for 30–60 s) and particle size control (2.5 –3 mm sieve openings) to balance starch gelatinization and PDI (Abdollahi et al., 2018; Svihus et al., 2024). However, the unique physicochemical profile of aged paddy rice—a strategic grain reserve with higher crude fiber (8.2–12.5 %) and lower starch digestibility —challenges these conventional processing paradigms (Müller et al., 2022). While its nutritional merits, including comparable energy (13.5–14.2 MJ/kg) and crude protein content (7.0–8.8 %) to corn, position it as a viable substitute (Kim et al., 2021; Tan et al., 2023), its processing limitations necessitate re-evaluating the strict particle size distributions (The optimal feed particle size for broilers aged 1–21 days ranges between 700 and 900 μm, while for broilers aged 21–42 days, it increases to 1000–1200 μm.) and conditioning protocols mandated by global integrators like Tyson Foods and BRF S.A.

The rationale for parameter escalation (SO: 2.0–2.5 mm; CTM: 180 s; CTP: 75–85 °C) stems from two industrial constraints: (1) the grinding-resistant coarse fiber husk is difficult to break down, requiring a smaller sieve opening (SO) to ensure the desired particle size. (Soponronnarit et al., 2008); (2) reduced starch availability demands extended thermal exposure (≥180 s at 80–85 °C) to achieve corn-equivalent gelatinization (Thomas and van der Poel, 2020). Recent innovations by Cargill and New Hope Group further validate that controlled coarse grinding (D50 = 800–1,000 μm) enhances gizzard function in high-fiber diets, supporting our hypothesis that strategic parameter escalation could mitigate paddy rice's limitations without compromising zootechnical performance.

Despite extensive research on aged paddy rice's nutritional impacts (Sanchez et al., 2019; Varzaru et al., 2020), critical gaps persist in understanding its processing dynamics. Existing studies predominantly focus on growth performance metrics, neglecting the interplay between feed structure (e.g., particle size distribution, pellet integrity) and nutrient utilization in high-fiber matrices. This study pioneers the utilization of aged rice (50 % substitution rate for corn) in feed formulations, addressing processing challenges associated with its high-fiber characteristics. Employing a factorial experimental design, we systematically investigated the interactive effects of three critical processing parameters: SO, CTP, and CTM on pellet quality, meat characteristics, and serum metabolic profiles. Our findings bridge the research gap in multi-parameter synergistic optimization for aged paddy rice-based feed processing. The established parameter optimization matrix provides technical support for refining feed formulations incorporating aged paddy rice, particularly in post-formulation adjustment of processing technologies.

## Materials and methods

### *Feed preparation and processing*

The feed preparation process includes grinding, batching, mixing, conditioning, pelleting, and drying. First, aged paddy rice, corn, and soybean meal were processed using a 9FQ-50B hammer mill (Beijing Tongyan Machinery Equipment Co., Ltd.), which is equipped with 24 symmetrical hammers and adjustable screens (2.0 mm and 2.5 mm). The materials were ground to a uniform particle size at a speed of 3200 rpm. With a 2.0 mm sieve, corn had a mean particle size of 464.13 ± 15.42 µm, while aged paddy rice had 503.56 ± 23.05 µm. Using a 2.5 mm sieve, corn had a mean particle size of 510.12 ± 8.73 µm, and aged paddy rice measured 574.98 ± 25.10 µm. After manual batching, the mixture was blended for 5 min using an SSHJ0.2 twin-shaft paddle mixer (Jiangsu Zhengchang Group), achieving a coefficient of variation for mixing uniformity ≤5 %.

The feed was then conveyed to the SJHS 0.2 conditioning system (Jiangsu Sibeide), where it was conditioned in a 0.2 m³ chamber with steam at 0.3–0.5 MPa pressure, while being mixed by a single-shaft paddle (20–40 rpm) for 90 s and 180 s. This process increased the moisture content of the feed from 12 % to 15–16 % and raised the temperature to 75 °C and 85 °C, respectively (conditioning temperature was controlled by adjusting steam pressure, while conditioning time was controlled by adjusting paddle speed). The conditioned feed was collected for moisture testing before entering the pelleting chamber.

The pelleting process was carried out using an SZLH200 × 40 ring die pellet mill (Jiangsu Zhengchang), which is equipped with a 200 mm diameter and 40 mm effective length ring die and a 150 mm roller. The die hole diameter was 3 mm, with a length-to-diameter ratio of 10:1. During operation, the gap between the roller and die was maintained at 0.2 mm, with a feed rate of 800–1000 kg/h, controlled by frequency conversion. A dynamic cutter was used to adjust the pellet length to 8–10 mm.

### *Birds and experimental design*

A total of 960 healthy, 1-day-old white-feathered broiler roosters were randomly assigned to 8 treatment groups, with each group consisting of 6 replicates of 20 chickens. The experiment lasted for 42 days and was divided into two phases: the first phase (days 1–21) and the second phase (days 22–42). All groups were fed with the same base feed formula, and the feed production followed a full factorial experimental design with eight different production processes based on combinations of grinding SO (2.0 mm and 2.5 mm), CTP (75 °C and 85 °C), and CTM (90 s and 180 s) (Table 1). Throughout the experiment, the broilers were housed in single-tier suspended cages, with free access to feed and water, and exposed to 16 h of light per day. The room temperature was gradually reduced by 2 °C each week, starting at 35 °C in the first week. The basic nutritional composition of both corn and aged paddy rice was analyzed (Table 2), and aged paddy rice replaced 50 % of the corn in the feed. Nutritional standards for the diets were based on guidelines for broiler pullets from the National Research Council (1994) (Table 3). Total energy and crude protein content were determined according to AOAC recommendations (1994), while other nutritional parameters were calculated values. The study was conducted at the experimental base of the Chinese Academy of Agricultural Sciences (CAAS), and all procedures were approved by the Animal Care and Use Committee of the Institute of Feed Research, CAAS.

**Table 1:** 2 × 2 × 2 full factorial experimental design for feed grinding and pelleting process parameters.

Group	Sieve opening	Conditioning temperature	Cinditioning time
CB-LLL	2.0 mm	75 °C	90 s
CB-LLH	2.0 mm	75 °C	180 s
CB-LHL	2.0 mm	85 °C	90 s
CB-LHH	2.0 mm	85 °C	180 s
CB-HLL	2.5 mm	75 °C	90 s
CB-HLH	2.5 mm	75 °C	180 s
CB-HHL	2.5 mm	85 °C	90 s
CB-HHH	2.5 mm	85 °C	180 s

**Table 2:** Nutrient content of aging paddy rice and corn(%).

Sample	Moisture	Crude protein	Crude ash	Crude fat	Crude fibre	Total starch	Amylopectin	Amylose
Aged paddy rice	10.08 ± 0.01	7.77 ± 0.02	3.27 ± 0.10***	2.06 ± 0.01	9.75 ± 0.21***	58.02 ± 2.10	84.47 ± 0.53***	15.53 ± 0.53
Corn	11.24 ± 0.04	7.74 ± 0.04	1.20 ± 0.00	3.56 ± 0.04***	1.80 ± 0.00	59.06 ± 0.94	77.89 ± 1.30	22.11 ± 1.39***

(1) The contents of amylopectin and amylose are expressed as their proportion in the total starch.

(2) Data in the same column with *P* < 0.001 are marked with ***.

**Table 3:** Composition and nutrient levels of experimental diet (%, air-dry basis).

Items	Grower phase (d 1 to 21)	Grower phase (d 22 to 42)
Ingredients		
Corn	27.90	31.20
Aged paddy rice	27.90	31.20
Soybean meal	33.49	26.59
Soybean oil	3.86	4.07
Corn protein powder 60 %	1.42	3.00
NaCl	0.32	0.32
CaHPO_4_	1.83	1.63
Stone powder	1.22	1.11
L-Lys	0.40	0.32
DL-Met	0.30	0.23
L-Threonine	0.01	0.00
Choline chloride	0.20	0.15
Premix ^1)^	0.13	0.17
Total	100	100
Nutrient levels^2)^		
ME (kcal/kg)	12.38	12.41
CP	21.000	18.500
Lys	1.300	1.100
Met	0.629	0.550
Met+Cys	0.950	0.850
Thr	0.820	0.724
Trp	1.274	0.238
Ca	0.950	0.850
TP	0.697	0.650
AP	0.450	0.420

(1) The premix provided the following per kg diets: vitamin A 12.00 kIU; vitamin B_1_ 4.0 mg; vitamin B_2_ 6.25 mg; vitamin B_6_ 6.0 mg; vitamin B_12_ 0.02 mg; vitamin D_3_ 4.0 kIU; vitamin E 20 IU; vitamin K 10.25 mg; Biotin 0.15 mg; Folic acid 1.65 mg; Pantothenic acid 15 mg; Nicotinic acid 50.00 mg; Cu 15 mg; Fe 90 mg; Mn 90 mg; Zn 60 mg; I 1.00 mg; Se 0.30 mg.

(2) In terms of nutrient levels, crude protein (CP) and metabolizable energy (ME) are measured values, while the remaining nutritional parameters are calculated values.

### *Physical pellet quality analysis*

5 kg of pellet feed from each group were collected using the tetrad sampling method and then sieved with a 3.150 mm analytical sieve (Eckhardt DIN 4188, Hann, Germany) for physical pellet quality analysis. The Pellet Durability Index (PDI) values were measured using an NHP100 PDI tester (Fahrenholz, 2012). Briefly, 500 g of intact pellets (free from dust) were placed in the PDI tester. The samples were tumbled at 50 rpm for 60 s in a sealed box, then removed, sieved, and weighed (m_1_). PDI was calculated using Eq. (1). Four parallel measurements were taken from the same feed batch to assess method accuracy; the average PDI value is reported for each treatment.

Pellet hardness was assessed using a texture analyser (TA.XT2i, Stable MicroSystems Co. Ltd, UK) equipped with a cylindrical probe (probe number 3) set to compression test mode (2 mm target distance and 1.5 mm/s speed). Following the method described by Svihus et al. (2004), 27 measurements were taken from pellets originating from the same batch to ensure analytical precision. These measurements were averaged to represent the pellet hardness of each treatment, rather than being treated as independent replicates for statistical analysis.

(1) $$PDI=\frac{m_{1}}{500}\times 100\%$$

### *Sample collection and characterization*

In the experiment, body weight (BW), average daily gain (ADG), average daily feed intake (ADFI), and feed conversion ratio (FCR) were measured for broilers in each cage at 21 and 42 days of age. ADFI and FCR values were adjusted to account for mortality. At the end of the experiment, one chicken was randomly selected from each replicate for blood collection, which was obtained from the wing vein using procoagulant vessels. Blood samples were left at room temperature for 4 h, then centrifuged at 3000 g for 15 min. The resulting serum samples were stored at −80 °C for later analysis. After blood collection, the selected chickens were euthanized by cervical dislocation. Trimmed body weights, gutted weights, pectoral muscle weights, thigh muscle weights, liver weights, and abdominal fat weights were recorded in accordance with the "Terminology and Statistical Measurement of Poultry Productive Performance in China" (NY/T823-2004). The left pectoral muscle and left leg muscle were then collected, with any fat tissue removed, and stored at −80 °C for subsequent analysis of meat quality traits. Additionally, the lengths and weights of the duodenum, jejunum, and ileum were recorded, with intestinal weights expressed as a percentage of BW.

### *Meat quality*

#### Drip loss

Drip loss was assessed by measuring the weight loss of pectoral muscle samples after refrigeration at 4 °C for 24, 48, and 72 h. Briefly, an 80 g sample (± 0.001 g) was taken from the left pectoral muscle, placed in a sealed container, and stored at 4 °C. The sample was weighed again at 24, 48, and 72 h. Drip loss was expressed as a percentage of the initial sample weight. For each replicate, triplicate measurements were taken to assess parallel variations within the same muscle sample, and the average of these measurements was used for statistical analysis.

#### Steaming loss

Steaming loss was determined by weighing pectoral muscle samples before and after cooking. The samples were cooked in a water bath at 80 °C until the internal temperature reached 70 °C. After cooking, the samples were cooled, stored at 4 °C for 12 h, and reweighed to calculate cooking loss as a percentage of the original weight. Again, each sample was measured three times to ensure method accuracy, and the mean value for each replicate was used for data analysis.

#### Tenderness

Tenderness was measured using a Texture Analyzer (TA.XT.plus, Stable Micro Systems, UK). From each cooked pectoral muscle, 10 strips (10 mm × 10 mm × 20 mm) were cut parallel to the muscle fibers. The Texture Analyzer operated under the following conditions: a crosshead speed of 1 mm/s, a trigger force of 5.0 g, and a working distance corresponding to 75 % strain. These 10 strip measurements were considered parallel measurements for the same replicate sample, and their average was used to represent the tenderness for that replicate.

#### Colour

The surface colour of the pectoral and leg muscles was measured with a calibrated CM-400 colorimeter (Konica Minolta, Ramsey, NJ). To minimize background interference, samples with a thickness of 2 cm were used. Colour values were measured based on the Commission Internationale de l'Illumination (CIE) system, reporting L*, a*, and b* values. L* represents lightness, ranging from 0 (black) to 100 (white), a* represents redness, ranging from −120 (green) to +120 (red), and b* represents yellowness, ranging from negative (blue) to positive (yellow) values. Each sample was measured three times for method consistency, and the average was recorded for statistical evaluation.

### Serum biochemistry

Serum total protein, albumin, and urea nitrogen levels were measured using a clinical chemistry auto-analyzer (Mindray BS-200 Chemistry Analyzer, Shenzhen, China). All assays were performed in duplicate for accuracy. Globulin levels were calculated by subtracting the albumin concentration from the total serum protein concentration.

### *Serum immunoglobulin*

Serum immunoglobulins (IgG, IgA and IgM) were quantified using a chicken immunoglobulin ELISA kit (Cusabio, Wuhan, China) according to the manufacturer's instructions.

### *Determination of antioxidant capacity*

The activities of glutathione peroxidase (GSH-Px), catalase (CAT), and superoxide dismutase (SOD) were evaluated using commercial assay kits provided by the Nanjing Jiancheng Bioengineering Institute (Nanjing, China). The results were calculated according to the manufacturer's instructions and expressed in units per gram of serum (U/g). Similarly, the malondialdehyde (MDA) content was determined using a commercial kit from the same manufacturer, with the results expressed in grams per liter of serum (g/L), following the manufacturer's guidelines.

### *Statistical analysis*

In this study, a completely randomized 2 × 2 × 2 factorial design was employed to evaluate the effects of three factors: two sizes of SO (2.0 mm and 2.5 mm), two sizes of CTP (75 °C and 85 °C), and two sizes of CTM (90 s and 180 s). The interactions among these three factors were also examined. All data were analyzed using the General Linear Model (GLM) procedure in SAS (2004, SAS 9.1, Cary, NC). Duncan's multiple range test was used for pairwise comparisons between groups. Statistical significance was defined as *P* < 0.05.

## Results

### *Physical pellet quality*

The hardness and PDI of the feed were measured to analyze the effects of SO, CTP, and CTM on the physical quality of feed pellets (Table 4). The results showed that 85 °C conditioning temperature significantly increased particle hardness (*P* = 0.023). A significant interaction affecting pellet hardness was observed between *SO* *×* *CTM* and *CTP × CTM* (*P* = 0.008 and *P* = 0.004, respectively). The CB-LHL group exhibited the highest hardness at 57.85 N, while the CB-HLL group showed the lowest hardness at 43.73 N. PDI was measured using the NHP100 PDI tester. SO, CTP, and CTM each had highly significant effects on PDI (*P* = 0.000). Specifically, an SO of 2.5 mm reduced PDI, while a CTP of 85 °C and a CTM of 180 s both increased PDI. Additionally, significant interactions were observed for PDI between *SO × CTP* (*P* = 0.000), *SO × CTM* (*P* = 0.046), *CTP × CTM* (*P* = 0.000), and *SO × CTP × CTM* (*P* = 0.000). The CB-LHH group exhibited the highest PDI at 87.94 %, while the CB-HLL group had the lowest PDI at 76.82 %. These findings suggest that conditioning temperature is the primary factor influencing pellet hardness, while screen opening, conditioning temperature, conditioning time, and their interactions significantly impact PDI, providing a basis for optimizing pellet production processes.

**Table 4:** Effects of Screen Opening (SO), Conditioning Temperature (CTP), and Conditioning Time (CTM) on Pellet Hardness and Pellet Durability Index (PDI) in diets with 50 % aged paddy rice replacing corn.

Groups	Items	
	Hardness, N	PDI, %
SO		
2 mm	49.46	84.40^a^
2.5 mm	47.77	81.32^b^
SEM	0.816	0.491
CTP		
75 °C	46.81^b^	81.15^b^
85 °C	50.41^a^	84.57^a^
SEM	0.809	0.472
CTM		
90 s	49.23	81.73^b^
180 s	48.00	83.99^a^
SEM	0.817	0.527
Inertaction		
CB-LLL	46.50	84.58^c^
CB-LLH	46.97	82.92^bc^
CB-LHL	57.85	82.14^bc^
CB-LHH	46.50	87.94^d^
CB-HLL	43.73	76.82^a^
CB-HLH	50.05	80.29^b^
CB-HHL	48.83	83.38^c^
CB-HHH	48.47	84.80^c^
SEM	0.784	0.044
*P*-value		
SO	0.284	0.000
CTP	0.023	0.000
CTM	0.435	0.000
*SO × CTP*	0.243	0.000
*SO × CTM*	0.008	0.046
*CTP × CTM*	0.004	0.000
*SO × CTP × CTM*	0.414	0.000

### *Growth performance*

ADFI, ADG, FCR, and BW of broilers were measured to assess the effects of sieve opening (SO), conditioning temperature (CTP), and conditioning time (CTM) on growth performance (Table 5). The results showed that an SO of 2 mm significantly increased ADFI from day 1 to day 21 (*P* = 0.017) and decreased FCR over the same period (*P* = 0.001). Although the 85 °C CTP significantly reduced body weight at day 21 (*P* = 0.038), this reduction was fully compensated by day 42 (*P* = 0.834). While CTP had limited effects on most growth performance indicators, a significant interaction between *CTP × CTM* was observed for BW at day 21 (*P* = 0.034). Additionally, an interaction between *SO × CTP* significantly influenced ADFI from day 21 to day 42 and from day 1 to day 42 (*P* = 0.021 and *P* = 0.020, respectively). CB-LHL group had the highest values for ADFI (113.72 g), ADG (71.13 g) from day 1 to day 42, and BW at day 42 (3030.00 g), while the CB-HHL group had the lowest ADG (67.53 g) and BW at day 42 (2890.00 g). Overall, these findings indicate that the CB-LHL group achieved the best final growth performance.

**Table 5:** Effects of Sieve Opening (SO), Conditioning Temperature (CTP), and Conditioning Time (CTM) on broiler performance in diets with 50 % aged paddy rice replacing corn.

Groups	Items											
	1∼21d					21∼42d				1∼42 d		
	Initial weight, g	ADFI, g	ADG, g	FCR	BW, g	ADFI, g	ADG, g	FCR	BW, kg	ADFI, g	ADG, g	FCR
SO												
2 mm	44.35	65.82^a^	52.17	1.26^a^	1144.02	158.18	87.14	1.83	2973.92	112.00	69.65	1.63
2.5 mm	44.39	64.80^b^	52.47	1.23^b^	1151.62	156.40	84.32	1.87	2922.31	110.60	68.39	1.61
SEM	0.132	0.208	0.174	0.004	4.171	1.155	1.168	0.021	23.500	0.609	0.577	0.010
CTP												
75 °C	44.43	65.53	52.69^a^	1.24	1156.28^a^	158.02	85.08	1.87	2942.98	111.77	68.89	1.63
85 °C	44.30	65.09	51.94^b^	1.25	1139.36^b^	156.56	86.38	1.82	2953.25	110.83	69.16	1.60
SEM	0.132	0.219	0.166	0.005	4.019	1.157	1.183	0.021	23.800	0.614	0.584	0.010
CTM												
90 s	44.52	65.44	52.18	1.25	1146.74	157.83	85.72	1.85	2946.88	111.64	68.95	1.62
180 s	44.22	65.19	52.45	1.24	1148.90	156.75	85.74	1.84	2949.35	110.97	69.10	1.61
SEM	0.130	0.221	0.174	0.005	4.206	1.159	1.186	0.021	23.800	0.616	0.584	0.010
Inertaction												
CB-LLL	44.65	66.18	51.73	1.28	1135.32	154.71	83.79	1.86	2890.00	110.44	67.76	1.63
CB-LLH	44.17	65.50	53.11	1.23	1167.70	157.72	87.12	1.83	3000.00	111.61	70.11	1.60
CB-LHL	44.45	66.18	52.57	1.26	1148.50	161.27	89.68	1.80	3030.00	113.72	71.13	1.60
CB-LHH	44.12	65.43	51.25	1.28	1124.56	159.01	87.97	1.81	2970.00	112.22	69.61	1.61
CB-HLL	44.53	65.66	52.60	1.25	1157.77	159.30	86.18	1.86	2970.00	112.48	69.39	1.62
CB-HLH	44.38	64.78	53.33	1.21	1164.33	160.34	83.24	1.94	2910.00	112.56	68.29	1.65
CB-HHL	44.43	63.72	51.82	1.23	1145.39	156.06	83.24	1.89	2890.00	109.89	67.53	1.63
CB-HHH	44.20	65.05	52.13	1.25	1139.00	149.91	84.62	1.78	2920.00	107.48	68.38	1.57
SEM	0.139	0.205	0.155	0.004	3.941	1.121	1.222	0.021	24.349	0.595	0.599	0.011
*P*-value												
SO	0.882	0.017	0.331	0.001	0.341	0.433	0.256	0.332	0.296	0.247	0.300	0.664
CTP	0.634	0.295	0.020	0.227	0.038	0.519	0.599	0.242	0.834	0.431	0.822	0.316
CTM	0.287	0.548	0.385	0.190	0.786	0.630	0.995	0.761	0.960	0.577	0.905	0.524
*SO×CTP*	0.976	0.336	0.443	0.784	0.807	0.021	0.400	0.714	0.355	0.020	0.339	0.518
*SO×CTM*	0.699	0.259	0.428	0.587	0.794	0.516	0.748	0.911	0.703	0.677	0.822	0.905
*CTP×CTM*	0.953	0.201	0.016	0.001	0.034	0.172	0.941	0.379	0.666	0.284	0.690	0.697
*SO×CTP×CTM*	0.835	0.171	0.072	0.712	0.176	0.831	0.344	0.166	0.225	0.970	0.231	0.112

### *Carcass yields*

After slaughter, the effects of SO, CTP, and CTM on broiler slaughtering yield, carcass yield, thigh muscle yield, and breast muscle yield were analyzed (Table 6). The results showed that there was no significant effect of SO, CTP and CTM on any of the indicators of Carcass Yields. Regarding interaction effects, a significant interaction was observed between *CTP × CTM* for leg muscle yield (*P* = 0.04), suggesting that the combination of conditioning temperature and time may influence leg muscle development.

**Table 6:** Effects of SO, CTP, and CTM on slaughter performance of broilers fed diets with 50 % aged paddy rice replacing corn.

Groups	Items			
	Slaughtering, %	Carcass, %	Leg muscle rate, %	Pectoral muscle rate, %
SO				
2.0 mm	91.14	78.42	19.50	30.47
2.5 mm	92.99	78.09	19.69	31.36
SEM	0.652	0.304	0.223	0.892
CTP				
750 °C	91.28	78.05	19.89	30.75
85 °C	92.84	78.46	19.70	31.09
SEM	0.656	0.304	0.431	0.900
CTM				
90 s	91.57	78.06	19.96	30.94
180 s	92.55	78.46	19.24	30.89
SEM	0.662	0.304	0.426	0.900
Inertaction				
CB-LLL	87.37	78.01	19.79	30.14
CB-LLH	91.94	78.2	19.99	29.07
CB-LHL	92.77	79.25	19.82	31.60
CB-LHH	92.46	78.21	18.43	31.08
CB-HLL	92.92	77.54	19.79	31.11
CB-HLH	92.89	78.47	20.00	32.67
CB-HHL	93.24	77.42	20.44	30.92
CB-HHH	92.9	78.94	18.55	30.75
SEM	0.654	0.314	1.681	3.665
*P*-value				
SO	0.165	0.609	0.64	0.34
CTP	0.240	0.523	0.18	0.71
CTM	0.462	0.527	0.09	0.95
*SO×CTP*	0.292	0.721	0.68	0.14
*SO×CTM*	0.381	0.195	0.78	0.42
*CTP×CTM*	0.327	0.796	0.04	0.75
*SO×CTP×CTM*	0.387	0.476	0.75	0.54

### *Digestive tract parameters*

The effects of SO, CTP, and CTM on digestive tract development in broilers were analyzed by measuring the relative weights and lengths of different digestive segments (Table 7). The results showed that SO had no significant impact on any measured indices (*P* > 0.05), although the group with a 2.5 mm SO exhibited a slight increase in gizzard weight ratio (from 1.17 % to 1.27 %). 85 °C CTP significantly reduced the ileum weight ratio (*P* = 0.040), and a 180 s CTM significantly shortened the duodenum length (*P* = 0.041).

**Table 7:** Effects of SO, CTP, and CTM on digestive tract development in 42-day broilers fed diets with 50 % aged paddy rice replacing corn.

Groups	Items							
	Gizzard weight ratio/%	Proventriculus weight ratio/%	Duodenum weight rate, %	Jejunum weight rate, %	Ileum weight rate, %	Duodenum length, cm	Jejunum length, cm	Ileum length, cm
SO								
2 mm	1.17	0.71	0.37	0.88	0.87	27.08	69.50	70.52
2.5 mm	1.27	0.65	0.38	0.85	0.85	27.29	69.51	69.73
SEM	0.034	0.028	0.010	0.022	0.036	0.481	1.046	1.154
CTP								
75 °C	1.23	0.67	0.39	0.90	0.94^a^	27.21	70.09	71.41
85 °C	1.21	0.69	0.37	0.83	0.79^b^	27.17	68.92	68.84
SEM	0.035	0.028	0.010	0.022	0.034	0.481	1.043	1.140
CTM								
90 s	1.24	0.67	0.38	0.87	0.90	28.19^a^	69.94	71.15
180 s	1.20	0.69	0.37	0.85	0.82	26.19^b^	69.07	69.10
SEM	0.034	0.028	0.010	0.022	0.036	0.458	1.044	1.145
Inertaction								
CB-LLL	1.21	0.68	0.42	0.99	1.08	29.33	71.75	75.08
CB-LLH	1.19	0.72	0.36	0.86	0.87	24.83	67.83	68.53
CB-LHL	1.07	0.63	0.37	0.86	0.82	28.25	69.17	67.83
CB-LHH	1.20	0.83	0.33	0.81	0.73	25.92	69.25	70.63
CB-HLL	1.30	0.74	0.37	0.89	0.86	27.33	71.09	71.92
CB-HLH	1.20	0.56	0.39	0.86	0.94	27.33	69.67	70.10
CB-HHL	1.37	0.65	0.38	0.75	0.85	27.83	67.75	69.75
CB-HHH	1.20	0.65	0.39	0.89	0.76	26.67	69.52	67.13
SEM	0.035	0.028	0.010	0.022	0.035	0.472	1.103	1.181
*P*-value								
SO	0.159	0.238	0.632	0.454	0.767	0.827	0.998	0.738
CTP	0.821	0.790	0.318	0.101	0.040	0.965	0.600	0.283
CTM	0.574	0.807	0.368	0.695	0.268	0.041	0.694	0.391
*SO×CTP*	0.472	0.807	0.307	0.727	0.466	0.965	0.793	0.999
*SO×CTM*	0.206	0.068	0.153	0.108	0.314	0.142	0.639	0.943
*CTP×CTM*	0.780	0.127	0.901	0.165	0.875	0.793	0.420	0.371
*SO×CTP×CTM*	0.422	0.939	0.826	0.557	0.293	0.383	0.927	0.289

### *Breast muscle meat quality*

Drip loss, cooking loss, and tenderness of broiler breast muscle were measured to analyze the effects of SO, CTP, and CTM on meat quality (Table 8). The results showed that 2.5 mm SO significantly reduced drip loss at 48 h (from 6.98 % to 6.49 %, *P* = 0.002), at 72 h (from 7.68 % to 7.11 %, *P* = 0.006), and cooking loss (from 10.33 % to 10.00 %, *P* = 0.018). CTP did not show a significant effect on any measured parameter (*P* > 0.05), although 85 °C CTP tended to improve drip loss at 24 h (*P* = 0.397), 48 h (*P* = 0.810), and 72 h (*P* = 0.501). Extending CTM from 90 s to 180 s significantly increased 24-hour drip loss (from 2.47 % to 4.29 %, *P* = 0.044) and cooking loss (from 9.7 % to 10.63 %, *P* = 0.001). In terms of interaction effects, there was a significant interaction between *SO × CTM* for 24-hour drip loss (*P* = 0.019) and between *SO × CTP* for cooking loss (*P* = 0.001). A three-way interaction among SO × *CTP × CTM* was significant for 48-hour drip loss, 72-hour drip loss, and cooking loss (*P* < 0.05). These results indicate that SO and CTM have the most substantial effects on moisture loss in broiler breast muscle, suggesting they play a crucial role in influencing meat quality attributes related to water retention.

**Table 8:** Effects of SO, CTP, and CTM on breast muscle quality in broilers fed diets with 50 % aged paddy rice replacing corn.

Groups	Items				
	Drip loss rate at 24 h, %	Drip loss rate at 48 h, %	Drip loss rate at 72 h, %	Cooking loss rate, %	Tenderness, N
SO					
2 mm	3.53	6.98^a^	7.68^a^	10.33^a^	18.30
2.5 mm	3.22	6.49^b^	7.11^b^	10.00^b^	20.05
SEM	0.461	0.506	0.546	0.352	1.092
CTP					
75 °C	3.49	6.79	7.47	9.74	19.43
85 °C	3.26	6.68	7.32	10.59	18.92
SEM	0.461	0.507	0.548	0.348	1.099
CTM					
90S	2.47^b^	5.77	6.34	9.70^b^	19.83
180S	4.29^a^	7.70	8.45	10.63^a^	18.52
SEM	0.442	0.487	0.525	0.525	1.096
Inertaction					
CB-LLL	1.60	4.53	4.69	9.09	18.44
CB-LLH	1.76	5.19	5.85	9.31	16.31
CB-LHL	1.77	5.11	6.07	10.36	20.40
CB-LHH	1.36	4.44	4.75	13.04	18.04
CB-HLL	1.11	6.60	6.83	8.49	19.48
CB-HLH	3.45	5.70	6.68	12.25	23.47
CB-HHL	1.56	5.01	5.50	8.56	20.98
CB-HHH	2.10	7.09	6.73	8.35	16.25
SEM	0.464	0.506	0.541	0.351	1.123
*P*-value					
SO	0.176	0.002	0.006	0.018	0.442
CTP	0.397	0.810	0.501	0.480	0.822
CTM	0.044	0.440	0.531	0.001	0.563
*SO × CTP*	0.630	0.980	0.300	0.000	0.301
*SO × CTM*	0.019	0.440	0.409	0.697	0.678
*CTP × CTM*	0.082	0.289	0.459	0.368	0.325
*SO × CTP × CTM*	0.362	0.009	0.014	0.001	0.350

### *Meat color*

Surface color of the breast and thigh muscles was measured using a calibrated CM-400 colorimeter to assess the effects of SO, CTP, and CTM on meat color, as shown in Table 9. The results indicated that SO, CTP, and CTM had no significant effects on the L* (lightness), a* (redness), and b* (yellowness) values of breast and thigh muscles (*P* > 0.05). However, the thigh muscle a* value in the 2.5 mm SO group was slightly higher (*P* = 0.054). A significant interaction was observed between SO × CTM for the thigh muscle b* value (*P* = 0.015).

**Table 9:** Effects of Sieve Opening (SO), Conditioning Temperature (CTP), and Conditioning Time (CTM) on breast muscle color in broilers fed diets with 50 % aged paddy rice replacing corn.

Groups	Pectoral muscle			Leg muscle		
	L^※^	a^※^	b^※^	L^※^	a^※^	b^※^
SO						
2 mm	69.65	3.48	−3.74	71.53	4.59	−1.79
2.5 mm	69.78	1.21	−3.74	71.09	5.69	−1.40
SEM	0.158	1.254	0.107	0.139	0.271	0.182
CTP						
75 °C	69.66	1.10	−3.81	71.34	5.13	−1.68
85 °C	69.76	3.60	−3.67	71.29	5.15	−1.51
SEM	0.158	1.252	0.106	0.143	0.283	0.184
CTM						
90 s	69.79	1.09	−3.81	71.51	4.88	−1.76
180 s	69.63	3.60	−3.67	71.12	5.40	−1.43
SEM	0.158	1.252	0.106	0.140	0.281	0.182
Inertaction						
CB-LLL	69.91	1.23	−3.58	72.21	4.25	−1.92
CB-LLH	69.03	1.12	−3.95	71.26	4.46	−2.18
CB-LHL	69.96	0.73	−3.68	71.76	5.19	−1.08
CB-LHH	69.68	10.86	−3.74	70.91	4.45	−1.96
CB-HLL	69.95	0.89	−4.03	70.83	5.20	−1.9
CB-HLH	69.76	1.16	−3.69	71.05	6.61	−0.71
CB-HHL	69.34	1.52	−3.93	71.24	4.87	−2.13
CB-HHH	70.07	1.27	−3.31	71.25	6.08	−0.87
SEM	0.162	1.257	0.109	0.137	0.278	0.176
*P*-value						
SO	0.680	0.371	0.989	0.115	0.054	0.281
CTP	0.760	0.327	0.504	0.866	0.972	0.635
CTM	0.625	0.324	0.551	0.160	0.356	0.351
*SO×CTP*	0.441	0.404	0.670	0.204	0.425	0.309
*SO×CTM*	0.196	0.326	0.121	0.071	0.165	0.015
*CTP×CTM*	0.249	0.339	0.502	0.921	0.611	0.703
*SO×CTP×CTM*	0.808	0.291	0.962	0.774	0.741	0.633

### *Serum biochemical parameters*

The effects of SO, CTP, and CTM on broiler serum biochemical indices are presented in Table 10. Results indicated that SO did not significantly affect total protein (TP), albumin (ALB), or globulin (GLB) (*P* > 0.05), though the 2.5 mm SO significantly increased blood urea nitrogen (BUN) levels (*P* = 0.000). CTP also had a significant effect on BUN (*P* = 0.017), with the 85 °C group showing higher BUN levels (1.85 g/L compared to 1.24 g/L). CTM significantly affected TP levels (*P* = 0.04), with the 180 s group exhibiting higher TP levels than the 90 s group (34.05 g/L vs. 32.54 g/L). A significant interaction between *CTP × CTM* was observed for BUN (*P* = 0.045), while a three-way interaction among *SO × CTP × CTM* was significant for GLB levels (*P* = 0.01). These findings suggest that sieve opening, conditioning temperature, and conditioning time influence protein and nitrogen metabolism in broilers, with notable effects on blood urea nitrogen and globulin levels.

**Table 10:** Effects of SO, CTP, and CTM on serum biochemical parameters in 42-day-old broilers fed diets with 50 % aged paddy rice replacing corn.

Groups	Items			
	TP, g/L	ALB, g/L	GLB, g/L	BUN, g/L
SO				
2 mm	32.87	20.55	12.32	1.02b
2.5 mm	33.72	20.56	13.16	2.08a
SEM	0.363	0.253	0.259	0.130
CTP				
75 °C	33.63	20.63	13.00	1.24b
85 °C	32.97	20.49	12.48	1.85a
SEM	0.365	0.253	0.264	0.145
CTM				
90 s	32.54b	20.26	12.28	1.56
180 s	34.05a	20.85	13.20	1.54
SEM	0.351	0.250	0.258	0.151
Inertaction				
CB-LLL	32.32	20.66	11.66	0.65
CB-LLH	34.25	20.69	13.57	0.92
CB-LHL	32.46	19.77	12.69	1.5
CB-LHH	32.46	21.09	11.37	1
CB-HLL	33.44	20.34	13.11	1.35
CB-HLH	34.51	20.83	13.68	2.05
CB-HHL	31.95	20.29	11.66	2.74
CB-HHH	34.99	20.8	14.19	2.18
SEM	0.355	0.265	0.238	0.123
*P*-value				
SO	0.238	0.981	0.086	0.000
CTP	0.354	0.790	0.278	0.017
CTM	0.040	0.274	0.060	0.936
*SO×CTP*	0.826	0.852	0.904	0.555
*SO×CTM*	0.447	0.873	0.192	0.704
*CTP×CTM*	0.990	0.542	0.509	0.045
*SO×CTP×CTM*	0.177	0.551	0.010	0.621

### *Serum Immunoglobulin*

Serum IgG, IgA, and IgM were quantified using chicken immunoglobulin ELISA kits to investigate the effects of SO, CTP, and CTM on broiler immune function (Table 11). The results showed that a 2.0 mm SO significantly increased serum IgM levels (*P* = 0.000). No significant effects were observed for CTP and CTM on IgG, IgA, or IgM levels (*P* > 0.05). Additionally, all interaction effects among SO, CTP, and CTM were non-significant for IgG, IgA, and IgM levels (*P* > 0.05).

**Table 11:** Effects of SO, CTP, and CTM on serum immunoglobulin levels in 42-day-old broilers fed diets with 50 % aged paddy rice replacing corn.

Groups	Items		
	IgG, g/L	IgA, g/L	IgM, g/L
SO			
2 mm	4.15	2.23	1.66
2.5 mm	4.12	2.20	1.60
SEM	0.017	0.015	0.007
CTP			
75 °C	4.12	2.22	1.63
85 °C	4.15	2.21	1.63
SEM	0.264	0.015	0.008
CTM			
90 s	4.13	2.23	1.63
180 s	4.15	2.21	1.63
SEM	0.015	0.015	0.008
Inertaction			
CB-LLL	4.17	2.25	1.67
CB-LLH	4.12	2.23	1.65
CB-LHL	4.12	2.23	1.68
CB-LHH	4.21	2.21	1.64
CB-HLL	4.10	2.23	1.59
CB-HLH	4.09	2.18	1.59
CB-HHL	4.12	2.20	1.60
CB-HHH	4.17	2.20	1.62
SEM	0.018	0.016	0.007
*P*-value			
SO	0.341	0.424	0.000
CTP	0.360	0.754	0.508
CTM	0.560	0.452	0.523
*SO×CTP*	0.756	0.888	0.512
*SO×CTM*	0.977	0.989	0.195
*CTP×CTM*	0.154	0.708	0.955
*SO×CTP×CTM*	0.508	0.694	0.698

### *Serum antioxidant indices*

The levels of MDA, SOD, CAT, and GSH-Px were measured to analyze the effects of SO, CTP, and CTM on antioxidant indices in broilers (Table 12). The results indicated that SO, CTP, and CTM did not have significant effects on any antioxidant indices (*P* > 0.05). However, CTM had a near-significant effect on CAT activity (*P* = 0.067), with the 90 s group showing slightly higher CAT activity (9.30 U/ml compared to 8.79 U/ml). These findings suggest that while the primary factors did not significantly influence antioxidant indices, there may be trends indicating potential interactions, particularly for CAT and GSH-Px activities, which could contribute to variations in broiler antioxidant capacity.

**Table 12:** Effects of SO, CTP, and CTM on serum antioxidant indices in 42-day-old broilers fed diets with 50 % aged paddy rice replacing corn.

Groups	Items			
	MDA, g/L	SOD, U/ml	CAT, U/ml	GSH-Px, U/ml
SO				
2 mm	4.61	113.42	9.27	988.77
2.5 mm	4.86	110.43	8.81	952.93
SEM	0.077	0.874	0.134	10.887
CTP				
75 °C	4.68	112.56	9.06	983.44
85 °C	4.80	111.29	9.02	958.26
SEM	0.078	0.897	0.138	11.048
CTM				
90 s	4.64	113.33	9.30	987.31
180 s	4.84	110.52	8.79	954.39
SEM	0.078	0.877	0.133	10.937
Inertaction				
CB-LLL	4.22	117.95	9.84	1040.31
CB-LLH	4.69	112.86	9.07	1002.61
CB-LHL	4.65	113.18	9.38	964.28
CB-LHH	4.89	109.71	8.80	947.88
CB-HLL	4.88	110.53	8.88	971.15
CB-HLH	4.92	108.92	8.47	919.70
CB-HHL	4.79	111.66	9.09	973.51
CB-HHH	4.85	110.59	8.82	947.38
SEM	0.078	0.871	0.135	10.695
*P*-value				
SO	0.118	0.093	0.095	0.102
CTP	0.451	0.468	0.878	0.246
CTM	0.202	0.114	0.067	0.132
*SO×CTP*	0.21	0.132	0.244	0.068
*SO×CTM*	0.325	0.403	0.547	0.785
*CTP××CTM*	0.747	0.758	0.76	0.589
*SO×CTP×CTM*	0.685	0.879	0.978	0.963

## Discussion

In this study, we investigated the effects of different processing parameters in a feed formula where 50 % of corn was replaced with aged paddy rice, examining impacts on feed quality, broiler growth, slaughter performance, digestive tract development, meat quality, and serum biochemistry. The significantly higher crude fiber content in aged paddy rice—more than four times that of corn (Chai et al., 2024)—markedly influenced feed processing characteristics. The coarse fiber is both resilient, with a lower density and specific heat, which makes it more challenging to handle during grinding and conditioning (Kirsten et al., 2016). This characteristic hinders effective steam penetration, resulting in lower conditioning temperatures for feed pellets, incomplete feed cooking, and ultimately, reduced digestion efficiency (Liao et al., 2017; Perera et al., 2021). Aiming at the challenge of high fiber characteristics of aged paddy rice on processing parameters, this study improved feed particle quality and nutrient metabolism of broilers by optimizing the combination of process parameters of crushing process (SO) and conditioning process (CTP, CTM).

Traditionally, feed industry experts prefer to adjust sieve opening size, conditioning parameters, and die compression ratios to achieve acceptable pellet hardness and PDI (Abadi et al., 2019). Consistent with our findings, 2.0 mm SO produces finer particles, increasing the specific surface area, which is more favorable for feed contact with steam. By increasing 85 °C CTP and extending 180 s CTM, the penetration steam into the feed particles is enhanced, which is beneficial to the starch gelatinization and protein denaturation in the feed. This combination of starch gelatinization and protein plasticization promotes particle adhesion. As the gelatinized starch cools, it forms a gel within the dispersed particle matrix, acting as a binder or ligand, ultimately strengthening pellet cohesion (Kannadhason et al., 2011; Cavalcanti and Behnke, 2005). Pellet hardness and PDI were significantly improved, with PDI reaching 87.94 % under the conditions of a 2.0 mm SO, 85 °C CTP, and 180 s CTM.

The SO plays a critical role in controlling feed particle size, significantly impacting broiler digestive efficiency and feeding behavior. Nir et al. (1995) indicated that larger feed particles can slow the passage of digestive fluids in the small intestine, enhancing peristalsis and nutrient utilization. Moreover, increasing particle size reduces feed surface area, which may impact digestion efficiency but can boost feed intake and improve gut health (Ege et al., 2019). This study demonstrates that 2.0 mm SO significantly raised ADFI in broilers aged 1 to 21 days to 65.82 grams, though FCR also increased to 1.26. In contrast, 2.5 mm SO, producing coarser particles, resulted in slightly lower feed intake (64.80 g) but improved FCR (1.23), indicating that coarser particles may enhance feed efficiency. Overall, 2.5 mm SO, 85 °C CTP, and 90 s CTM optimized broiler growth performance. These results align with findings from other studies. For instance, Chewning et al. (2012) compared broiler weight gain using ground corn with coarse (600 μm) and fine (300 μm) particle sizes. They found that finer particles significantly improved growth performance during the early phase (days 1-14), while the growth performance difference dissipated in the later phase (days 15-21). This may be due to the immature development of the broiler digestive system in early stages, where overly large particles could limit daily feed intake. However, as broilers mature, their digestive systems develop, and the gizzard becomes adapted to processing coarser particles, with coarse particles stimulating gizzard development and encouraging feeding behavior, ultimately enhancing growth performance.

Pelleting is a critical step in feed formation, with pelleting mills typically equipped with a conditioning system to aid this process (Cutlip et al., 2008). In the conditioner, thorough steam contact with the feed mash, which raises CTP. Under high-temperature and high-moisture conditions, disulfide bonds in proteins may break, improving protein digestibility through denaturation and enhancing protease activity, while also facilitating starch gelatinization. Extended retention time in the conditioner enables more even moisture and heat distribution within the feed, with gelatinized starch, which has strong adhesive properties, ultimately leading to improved pellet quality (Abdollahi et al., 2020; Boltz et al., 2020). In this study, 85 °C CTP and 180 s CTM proved beneficial for enhancing pellet quality, including hardness and PDI. Consistent with our findings, Briggs et al. (1999) demonstrated that extending retention time in the conditioner increased PDI by an average of 4.5 %. Despite the slight adverse effects of the 85 °C CTP on early growth performance (days 1–21), where ADG dropped from 52.69 g/day to 51.94 g/day, and BW decreased from 1156.28 g to 1139.36 g, overall growth performance over the full trial period (days 1–42) was better at 85 °C compared to 75 °C. Specifically, ADG increased from 68.89 g/day to 69.16 g/day, and BW at 42 days increased from 2942.98 g to 2953.25 g. This is in line with findings by Loar et al. (2014), who observed that raising conditioning temperature from 75 °C to 85 °C affected broiler utilization of various amino acids (such as lysine, cysteine, methionine), nutrients, and energy. Higher temperatures with prolonged conditioning times, however, may exacerbate negative effects on heat-sensitive dietary components. To some extent, reduced feed digestibility could be associated with the loss of additives, including enzymes, vitamins, and synthetic amino acids (Amerah et al., 2011; Amerah and Ravindran, 2009). In the finishing phase, broilers have higher energy demands and are less dependent on nutritional additives, making the adverse effects of high temperatures on heat-sensitive components less impactful on late-stage growth performance.

Shi et al. (2017) reported that when broilers were fed diets with corn ground to three different particle sizes (232 μm, 319 μm, and 380 μm), there was no impact on carcass yield. Similarly, Rezaeipour and Gazani (2014) found that feed particle size variations did not affect the relative weight of the thighs. In the present study, SO size, CTM, and CTP showed no significant differences in carcass yield or characteristics. However, 2.5 mm SO tended to increase slaughter yield, thigh muscle yield, and breast muscle yield. Additionally, 85 °C CTP resulted in a higher slaughter yield, carcass yield, and thigh muscle yield. This may be due to the increased feed particle size, which enhances chewing and digestion capacity, thereby stimulating muscle tissue development (Xu et al., 2015). Furthermore, the higher CTP may improve feed gelatinization and cooking, enhancing nutrient utilization and promoting muscle growth.

Promoting gizzard development is a nutritional strategy that can be achieved by controlling the feed particle size. Nir et al. (1994) reported a positive correlation between gizzard weight and particle size. In this study, 2.5 mm SO increased the relative weight of the gizzard, probably because larger feed particles stimulated masticatory behavior, enhanced mechanical grinding and digestion of the gizzard and gastric sinuses, and promoted the development of these organs (Lv et al., 2015). In addition, 85 °C CTP significantly reduced the relative weight of the ileum, and 180 s CTM significantly reduced duodenal length. This may be due to the fact that higher CTP and longer CTM promote the pasting and steaming of nutrients in the feed, which reduces the digestive load on the gastrointestinal tract and contributes to better nutrient absorption in broilers (Muramatsu et al., 2014; Liermann et al., 2019).

Research on the effects of SO, CTP, and CTM on meat quality in basal diets replacing 50 % corn with aged paddy rice is limited. In this study, 2.5 mm SO significantly reduced drip loss in breast muscle at both 48 h and 72 h, as well as cooking loss (Attia et al., 2014). This suggests that larger particle sizes of feed help improve meat water-holding capacity, with reduced moisture loss contributing to better texture, allowing the meat to retain a desirable mouthfeel during cooking. However, 180 s CTM significantly increased 24 h drip loss and cooking loss, while reducing shear force, indicating that prolonged CTM (180 s) may lead to a relatively looser meat texture, impacting water retention and tenderness. As for meat color, no significant differences were observed across processing parameters; however, the 2.5 mm SO slightly increased leg muscle redness (a*), showing some variation.

BUN reflects the efficiency of protein catabolism and nitrogen utilization. Larger particle sizes, combined with higher tempering temperatures, enhance protein-driven energy metabolism, directly linking processing adjustments to nitrogen metabolism. Specifically, larger particles (2.5 mm SO) reduce the degree of starch gelatinization by reducing the surface area through which steam penetrates, thus limiting the availability of energy. This forces broilers to obtain energy by metabolizing protein in the feed, increasing amino acid deamination and urea production (Singh et al., 2021). Additionally, 85 °C CTP can promote protein denaturation and improve digestibility, but may also accelerate protein turnover (Karlsson et al., 2012). The results of these interactions are consistent with Chuang et al. (2020), who studied the elevated BUN with coarse particle size in broilers fed a high-fiber diet.

In contrast, finer particles (such as 2.0 mm SO) increased the viscosity of chyme and slowed intestinal passage rates, which may have increased antigen exposure in gut-associated lymphoid tissue, stimulating IgM production. IgM is the first antibody in mucosal immunity (Li et al., 2016). IgG/IgA did not change significantly, suggesting local immune activation rather than systemic inflammation. Particle size directly regulates gut physiology because finer feed physically alters digestive properties, which in turn triggers an immune response. This is consistent with a study by Novotný et al. (2023) on the effect of particle size on intestinal morphology and immune markers.

Regarding antioxidant capacity, although no significant effects on MDA, SOD, CAT, and GSH-Px were observed, the effect of 90 s CTM on CAT activity was nearly significant, suggesting that shorter CTM may help maintain higher antioxidant enzyme activity. Nevertheless, longer CTM (180 s) may affect heat-sensitive antioxidants (vitamins or enzymes) through mild high temperatures, leading to decreased CAT activity. This result aligns with the study by Yang et al. (2020) on the stability of antioxidant systems under moderate processing conditions. Although the direct effects are limited, certain interactions (e.g., the effect of SO × CTP on GSH-Px, *P* = 0.068) suggest that processing parameters subtly influence the REDOX equilibrium through synergistic interactions.

Based on the close relationship between growth performance, meat quality, and the economic benefits of the poultry industry, this study recommends the use of a combination of 2.5 mm SO, 75 °C CTP, and 90 s CTM in the feed formulation, where 50 % of the corn is replaced by aged rice. This combination provides effective technical guidance for feed processing with high-fiber raw materials, successfully balancing pellet quality, broiler production performance, and meat quality.

## Conclusion

The combination of 2.5 mm SO, 75 °C CTP, and 90 s CTM emerged as the optimal configuration, balancing feed processing efficiency, broiler growth performance, and meat quality.

## Author's contributions

Formal analysis, JQZ; methodology, YS and XL; software, JGL and CZ; supervision, MQW; writing—original draft preparation, JQZ and XHS; writing—review and editing, JQZ; project administration, YCQ and JY. All authors read and approved the final manuscript.

## Ethics approval

All experimental procedures were reviewed and approved by the Animal Care and Use Committee at the Institute of Animal Science, Chinese Academy of Agricultural Sciences.

## Consent for publication

Not applicable.

## Availability of data and materials

The data analyzed during the current study are available from the corresponding author on reasonable request.

## Declaration of competing interest

The authors declare that they have no conflict of interest.
